# SIRT1-Mediated Redox and Senescence Regulation in Cancer: Mechanisms and Therapeutic Implications

**DOI:** 10.3390/antiox14091076

**Published:** 2025-09-02

**Authors:** Yejin Son, Minyeong Han, Xuefeng Wu, Yoon-Seok Roh

**Affiliations:** 1 College of Pharmacy and Medical Research Center, Chungbuk National University, Cheongju 28160, Republic of Korea; yjson2402@chnu.ac.kr (Y.S.); myone0306@chnu.ac.kr (M.H.); 2 Department of Immunology and Microbiology, Shanghai Institute of Immunology, School of Medicine, Shanghai Jiao Tong University, Shanghai 200025, China; xuefengwu@shsmu.edu.cn; 3 Hongqiao International Institute of Medicine, Shanghai Tongren Hospital, School of Medicine, Shanghai Jiao Tong University, Shanghai 200025, China

**Keywords:** SIRT1, redox signaling, cellular senescence, reactive oxygen species, cancer therapy

## Abstract

Silent information regulator type 1 (SIRT1), a NAD^+^-dependent deacetylase, is a central regulator of cancer cell adaptation to oxidative stress and senescence. By deacetylating redox-sensitive transcription factors, such as p53, FOXOs, PGC-1α, and NF-κB, SIRT1 suppresses apoptosis, delays senescence, enhances mitochondrial function, and attenuates pro-inflammatory senescence-associated secretory phenotypes. These mechanisms collectively promote tumor progression and contribute to resistance to therapy. Reactive oxygen species (ROS), long regarded as damaging byproducts, are now recognized as critical modulators of cancer biology. Although moderate ROS levels drive oncogenic signaling, excessive ROS accumulation triggers DNA damage, oxidative stress, and senescence. To survive these hostile conditions, cancer cells reinforce antioxidant defenses and exploit the NAD^+^–SIRT1 axis to maintain redox balance and evade senescence. The objective of this review was to provide an integrated framework linking SIRT1-mediated deacetylation to redox regulation and senescence control in cancer. We synthesized mechanistic insights into SIRT1 interactions with its substrates, highlighted cancer type-specific functions in ovarian, breast, liver, lung, and gastrointestinal malignancies, and critically evaluated the dual role of SIRT1 as both a longevity factor and an oncogenic driver. Finally, we explored the therapeutic implications of the pharmacological inhibition of SIRT1 as a strategy to restore senescence, increase ROS vulnerability, and overcome therapy resistance. This synthesis underscores the potential of the SIRT1–redox–senescence axis as a promising target in precision oncology.

## 1. Introduction

Cancer cells establish a sophisticated redox signaling network that enables them to thrive in microenvironments dominated by oxidative stress [1]. Reactive oxygen species (ROS), once regarded as metabolic byproducts, are now recognized as pivotal regulators of signaling cascades, genomic integrity, and disease progression. Although moderate ROS levels sustain tumor-promoting pathways, excessive ROS accumulation induces DNA damage, oxidative stress, and senescence [2]. To counter these threats, cancer cells reinforce their antioxidant defenses and reprogram redox-sensitive transcriptional networks, thereby evading senescence and apoptosis [2,3]. This enhanced antioxidant capacity constitutes the central mechanism of resistance to chemotherapy and radiotherapy, both of which rely on ROS-induced cytotoxicity [3,4]. In addition to oncology, ROS and oxidative stress are fundamental to the onset and progression of non-cancerous disorders, including cardiovascular, neurodegenerative, and metabolic diseases, underscoring their universal pathophysiological relevance [5].

The broad involvement of ROS in both malignant and non-malignant contexts highlights the necessity for master regulators that coordinate cellular responses to redox imbalances. Among the central regulators of these adaptive responses is the sirtuin family (silent information regulator type 1 [SIRT1]–SIRT7), a group of NAD^+^-dependent deacetylases and ADP-ribosyltransferases that orchestrate metabolism, inflammation, redox balance, and aging [6]. The field traces its origins to the discovery of Sir2 (Silent Information Regulator 2) in yeast, which was first recognized for extending lifespans under caloric restriction by stabilizing chromatin and preventing genomic instability via histone deacetylation [7]. In mammals, Sir2 has evolutionarily expanded into seven homologs (SIRT1–SIRT7), each with distinct subcellular localization: SIRT1, SIRT6, and SIRT7 in the nucleus; SIRT2 in the cytoplasm; and SIRT3–5 in the mitochondria [8]. Among these, SIRT1 retains the closest structural and functional resemblance to Sir2 and is the most intensively studied isoform, functioning as a master regulator of stress resistance, redox homeostasis, and longevity [9].

Physiologically, SIRT1 safeguards genomic integrity, mitigates oxidative stress, and delays senescence. However, these protective mechanisms are hijacked in cancer to promote malignant survival [10]. By deacetylating redox-sensitive transcription factors, such as p53, FOXOs, PGC-1α, and NF-κB, SIRT1 suppresses apoptosis, evades senescence, enhances mitochondrial resilience, and restrains the pro-inflammatory senescence-associated secretory phenotypes (SASP) [5,9,10,11,12,13]. Through these mechanisms, SIRT1 not only supports tumor progression but also fosters therapeutic resistance, making it a promising target for pharmacological inhibition.

This review integrates the mechanistic perspectives on how SIRT1-mediated deacetylation enables cancer cells to maintain redox homeostasis and evade senescence. Focusing on key redox-sensitive transcription factors, including p53, the FOXO family, PGC-1α, and NF-κB, we systematically summarize the impact of SIRT1 deacetylation on cancer cell survival, proliferation, and tumor microenvironment remodeling. Furthermore, this review underscores the coexistence of unresolved mechanistic uncertainties and therapeutic opportunities, while highlighting the dual identity of SIRT1 as both a longevity factor and an oncogenic driver, offering a novel framework that links redox biology, cancer adaptation, and therapeutic resistance [11].

## 2. Acetylation and Deacetylation in Cancer Epigenetics

Cancer cells are constantly exposed to harsh environmental stressors, such as oxidative stress, genotoxic damage, metabolic dysregulation, and nutrient deprivation. To survive and maintain cell proliferation under these conditions, cells activate highly controlled transcriptional programs that govern gene expression and protein functions. Among the key regulatory mechanisms involved, reversible protein acetylation and deacetylation are central to transcriptional and signaling modulation. These post-translational modifications influence both histone and non-histone substrates, playing essential roles in transcription, DNA repair, cell cycle control, senescence, and apoptosis [12]. This section provides an overview of the biochemical mechanisms and biological significance of acetylation and deacetylation in cancer, serving as a prelude to the detailed discussion on NAD^+^-dependent SIRT1 deacetylase functions in tumor adaptation [13].

### 2.1. Molecular Mechanisms of Acetylation

Protein acetylation is a key post-translational modification that regulates numerous aspects of protein function and gene expression [14]. This process involves the covalent transfer of an acetyl group from acetyl-CoA to specific sites on proteins, most commonly to either the α-amino group at the N-terminus or the ε-amino group of lysine residues [15].

Acetylation at the N-terminal α-amino group, referred to as Nα-acetylation, generally occurs co-translationally during protein synthesis [16]. This modification is irreversible and primarily affects protein stability and degradation, thereby influencing the overall half-life of proteins within the cell. Nε-acetylation, which targets internal lysine residues, is a reversible process that dynamically modulates protein behavior. This type of acetylation plays a critical role in controlling protein–protein interactions, subcellular localization, DNA-binding activity, and the transcriptional potential of various factors. These acetylation reactions are catalyzed by lysine acetyltransferases, a diverse group of enzymes that act on both histone and non-histone substrates [17]. Some of the well-known KATs include enzymes that target histones H3 and H4 to promote chromatin relaxation and transcriptional activation, while others acetylate key transcription factors, such as p53, FOXO1, and NF-κB, thereby influencing cellular responses to oxidative stress and DNA damage [18]. In addition to gene regulation, certain acetyltransferases contribute to genome stability and DNA repair by acetylating the components of the DNA damage response machinery. Through these multifaceted functions, acetylation provides cells with a versatile mechanism to finely tune physiological processes, adapt to environmental stresses, and promote survival and proliferation in pathological contexts, such as cancer [4].

### 2.2. Functional Roles of Acetylation in Cancer Cell Adaptation

**Figure 1:**
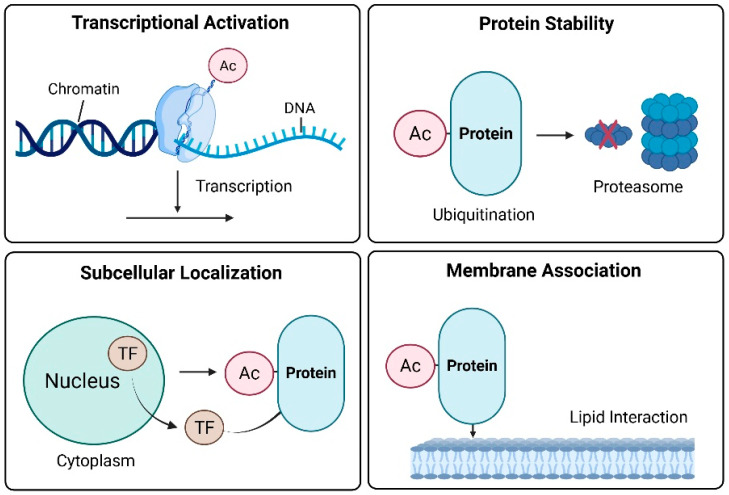
Functional roles of acetylation in cancer cell adaptation. The red X indicates protein degradation.

Cancer cells survive oxidative stress, DNA damage, metabolic imbalances, and immune challenges by precisely regulating adaptive pathways via protein acetylation [19]. In addition to its classical role in epigenetic regulation, acetylation dynamically alters the activity, stability, localization, and interactions of transcription factors and signaling proteins. This multifaceted control enables cancer cells to reprogram their fate and survive under hostile conditions. Acetylation enables diverse adaptive mechanisms in cancer cells [19]. These functions can be broadly categorized as transcriptional regulation, protein stability, and subcellular localization (Figure 1).

#### 2.2.1. Transcriptional Activation

Lysine acetylation of histone tails neutralizes their positive charge, thereby loosening the chromatin structure and enhancing the accessibility of the transcriptional machinery to DNA [20]. Chromatin decondensation facilitates the transcriptional activation of stress-responsive genes. Notably, acetylation is not limited to histones. Non-histone transcription factors, including p53, FOXO, and NF-Κb, are dynamically regulated by acetylation, linking external stress cues to gene expression programs. For example, p53 is acetylated at residue K382 by CBP/p300 in response to DNA damage and oxidative stress. This modification enhances the DNA-binding affinity of p53 and promotes the transcription of pro-apoptotic and cell cycle arrest genes, such as *p21^CIP1*, *PUMA*, and *BAX* [21]. Conversely, deacetylation by SIRT1 represses these transcriptional outputs and supports tumor cell survival [22]. FOXO1 acetylation increases its binding to the promoters of antioxidant enzymes (e.g., superoxide dismutase 2 (*SOD2*) and *catalase*), thereby enhancing cellular antioxidant capacity [23]. Similarly, NF-κB-dependent expression of immunomodulatory and pro-inflammatory genes relies on their acetylation status, which contributes to tumor immune evasion and chronic inflammation within the tumor microenvironment [24].

#### 2.2.2. Protein Stability

Acetylation frequently competes with ubiquitination at lysine residues, thereby preventing proteasome-mediated degradation and prolonging the half-lives of proteins critical for tumor progression. Oncoproteins, such as c-Myc and HIF-1α, are stabilized via acetylation, enabling their persistent oncogenic activity [25,26]. Acetylation can be used to mark proteins for degradation in a context-dependent manner. For example, E2F1 acetylation by p300 paradoxically reduces its stability and enhances pro-apoptotic signaling [27]. This dual nature underscores the nuanced and context-dependent role of acetylation in tumor biology.

#### 2.2.3. Subcellular Localization

The acetylation status of transcription factors determines their subcellular distribution. Under oxidative stress, FOXO3 is acetylated and exported from the nucleus to the cytoplasm, repressing its transcriptional activity [28]. This spatial exclusion blunts the expression of apoptotic and senescence-associated genes, favoring tumor cell persistence [29]. In contrast, the acetylation of NF-κB enhances its nuclear retention and prolongs the transcription of inflammatory mediators, contributing to a protumorigenic microenvironment [30]. Thus, localization dynamics shaped by acetylation serve as an adaptive switch that regulates cancer cell fate in response to microenvironmental cues [31].

#### 2.2.4. Membrane Association

Several signaling proteins and adaptors require membrane association for activation, a process governed in part by electrostatic interactions involving lysine residues. Acetylation neutralizes these residues, potentially disrupting protein–lipid interactions and altering subcellular localization [32]. Proteins, such as Ras and AKT, undergo acetylation-dependent modulation of membrane binding and signaling kinetics [33]. These changes affect the amplitude and duration of PI3K-AKT signaling, influencing metabolic reprogramming and the proliferative capacity of cancer cells. Taken together, acetylation acts as a central regulatory node that integrates transcriptional control, protein turnover, intracellular localization, and membrane signaling [19]. Through these multifaceted functions, acetylation enables cancer cells to dynamically adapt to environmental stressors and sustain malignant progression. Beyond proliferative signaling, acetylation-dependent modulation of PI3K-AKT-mTOR activity intersects with redox metabolism by enhancing glucose uptake and NADPH production, thereby reinforcing antioxidant defenses in tumor cells. This highlights the role of acetylation-mediated membrane association as a metabolic redox adaptation mechanism that supports malignant growth.

### 2.3. Deacetylation as a Reversible Transcriptional Control Mechanism

Acetylation is an inherently reversible process; therefore, deacetylation serves not only as a counteracting mechanism but also as a crucial axis for transcriptional repression and the regulation of protein function [23]. Deacetylases can be broadly divided into two major classes. The first comprises Zn^2+^-dependent histone deacetylases (HDACs), which promote transcriptional repression by compacting chromatin structure. The second category includes NAD-dependent sirtuins, which couple their activity to the cellular metabolic state and finely tune gene expression, stress responses, and signaling pathways [24]. Thus, although both HDACs and sirtuins mediate deacetylation, they exhibit distinct modes of action and operate in different biological contexts.

#### 2.3.1. Zn^2+^-Dependent HDACs

Zn^2+^-dependent HDACs represent the classical family of deacetylases that directly induce transcriptional repression through chromatin remodeling. They remove acetyl groups from histone tails by associating with transcriptional corepressor complexes, thereby reducing DNA accessibility [17]. This process can suppress the expression of tumor suppressor genes or, conversely, reinforce oncogenic signaling pathways. Class I HDACs (HDAC1, HDAC2, and HDAC3) primarily function in the nucleus, where they form complexes with corepressors, such as CoREST and NuRD, to mediate chromatin condensation and transcriptional repression. These actions enhance cancer cell proliferation, dedifferentiation, and resistance to apoptosis [25,26]. In contrast, Class II HDACs, such as HDAC6, preferentially target non-histone substrates and regulate cytoskeletal dynamics and stress granule formation. Consequently, they promote cell motility and invasiveness and play critical roles in cancer cell metastasis [26].

#### 2.3.2. NAD^+^-Dependent Sirtuins

Sirtuins represent a distinct class of NAD-dependent deacetylases that function as metabolic sensors linking the cellular energy status to transcriptional and stress response programs [34]. Among them, SIRT1 is the most extensively studied and is particularly relevant to cancer biology, as it regulates redox-sensitive transcription factors, such as p53, FOXO proteins, and NF-κB [35]. Through these interactions, SIRT1 fine-tunes the antioxidant responses, apoptosis, and senescence, thereby contributing to genomic stability and proliferative capacity under stress. Thus, sirtuin-mediated deacetylation provides a reversible and energy-dependent mechanism through which cancer cells dynamically adjust their stress signaling and survival pathways.

## 3. NAD^+^/SIRT1 Axis and Redox Control in Cancer

### 3.1. NAD^+^ Metabolism and SIRT1 Activation

SIRT1 is a NAD^+^-dependent deacetylase that plays a central role in cellular metabolism, aging, and survival. NAD^+^ serves as both a cofactor and direct substrate for SIRT1 activity [30]. During the deacetylation of lysine residues on target proteins, NAD^+^ is cleaved into nicotinamide, O-acetyl-ADP-ribose, and acetate [31]. NAD^+^ is regenerated in cells in limited amounts; therefore, its availability is a key determinant of SIRT1 enzymatic activity. In cancer cells, the NAD^+^–SIRT1 signaling pathway contributes to tumor survival by regulating redox homeostasis and suppressing cellular senescence [32]. When NAD^+^ levels are maintained or elevated, SIRT1 activity is enhanced, which inhibits senescence-related pathways and promotes cancer cell proliferation and survival [33]. This regulatory axis can be further strengthened by supplementation with NAD^+^ precursors, such as nicotinamide riboside (NR) or nicotinamide mononucleotide (NMN), which elevate intracellular NAD^+^ levels and consequently augment SIRT1 activity, reinforcing redox balance and resistance to senescence in cancer cells [34].

Beyond salvage and precursor supplementation pathways, nicotinamide N-methyltransferase (NNMT) is a master regulator of the NAD^+^-SAM-SIRT1 metabolic axis [36]. NNMT catalyzes the methylation of nicotinamide to 1-methylnicotinamide (1-MNA) using S-adenosylmethionine (SAM) as the methyl donor [37]. Through this reaction, NNMT simultaneously depletes NAD^+^ precursors and consumes SAM, thereby reducing intracellular NAD^+^ availability, altering the methylation potential, and indirectly modulating NAD^+^-dependent enzymes, including SIRT1 [38]. Ulanovskaya et al. demonstrated that NNMT rewires the cellular methylation capacity by draining SAM pools, thus coupling NAD^+^ salvage metabolism with epigenetic regulation [38]. Elevated NNMT expression has been consistently reported in multiple cancers, where it enhances tumorigenicity, drives epigenetic reprogramming, promotes redox imbalance, and contributes to drug resistance [39]. NNMT should thus be regarded not as a solitary oncogenic effector but as a pivotal metabolic checkpoint that indirectly regulates SIRT1 activity, positioning it at the intersection of NAD^+^ and SAM metabolism in cancer [40].

**Figure 2:**
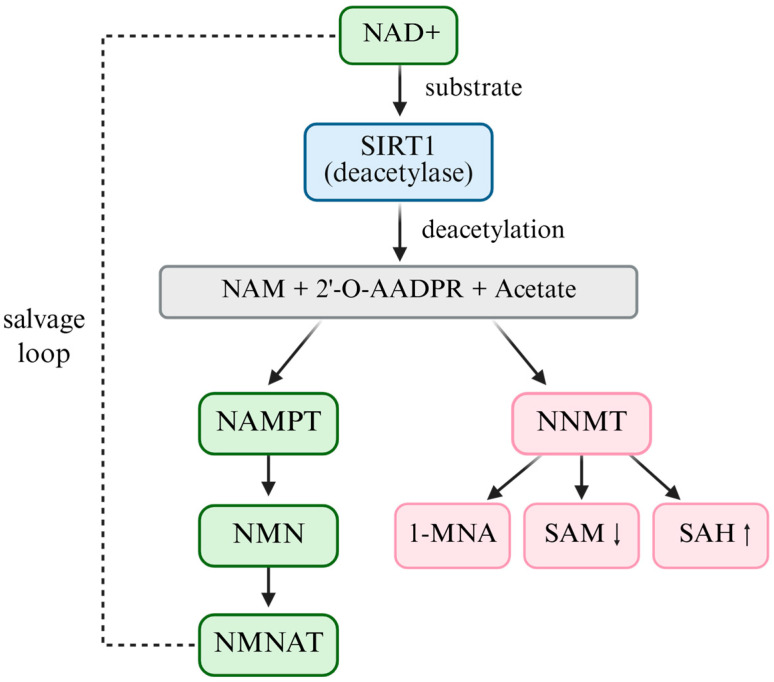
The NAD^+^–SIRT1–NNMT metabolic axis.

In addition to enzymatic regulators, such as NNMT, intracellular NAD^+^ concentrations fluctuate dynamically in response to metabolic stress, including energy deficiency, oxidative stress, and nutrient scarcity [41]. Under such conditions, NAD^+^ levels may transiently increase, leading to enhanced SIRT1 activation. Cancer cells exploit this adaptive mechanism by upregulating the NAD^+^–SIRT1 axis to reinforce antioxidant defenses, resist oxidative damage, and evade senescence [35]. Given its central role in regulating both NAD^+^ and SAM metabolism, therapeutic targeting of NNMT has gained attention as a strategy to restore redox balance and epigenetic integrity in tumors [42,43]. Several small-molecule NNMT inhibitors have been developed and are currently under preclinical investigation, demonstrating promising efficacy in overcoming chemoresistance and suppressing tumor progression [44,45]. (Figure 2)

### 3.2. Redox-Sensitive Deacetylation Activity of SIRT1

SIRT1 exerts its deacetylase activity in a manner that is highly sensitive to the cellular redox environment [46]. SIRT1 function is dependent on the availability of NAD^+^; therefore, its activity dynamically responds to metabolic and oxidative stress conditions [47]. This suggests that SIRT1 is a redox-sensitive regulator that integrates environmental signals into adaptive cellular responses. SIRT1 promotes cancer cell survival and adaptation under stressful conditions by deacetylating various transcription factors and metabolic regulators. Representative targets include p53, FOXO family transcription factors, PGC-1α, and NF-κB [46]. By modulating the activity of these factors in a redox-dependent manner, SIRT1 enables cancer cells to sustain growth and resist metabolic and oxidative stress.

### 3.3. SIRT1 Substrates Involved in Redox Homeostasis

**Figure 3:**
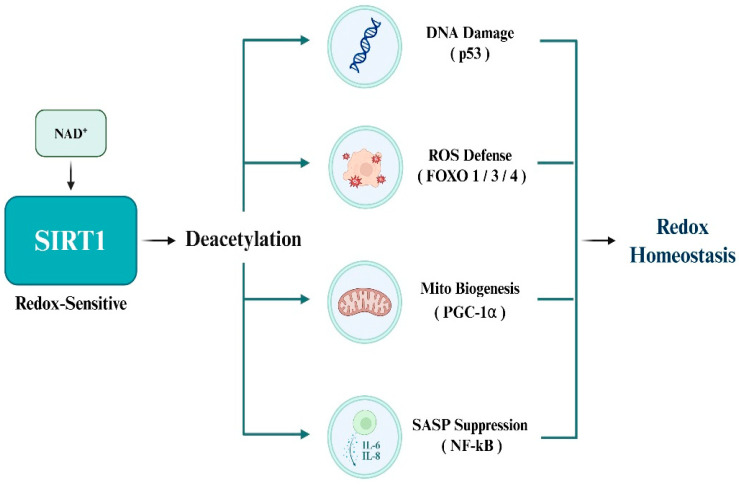
Redox homeostasis via SIRT1-mediated deacetylation.

SIRT1 deacetylates a range of transcription factors involved in antioxidant defense, mitochondrial regulation, and inflammatory signaling to maintain redox homeostasis under oxidative stress. The best-characterized redox-sensitive substrates include p53, members of the FOXO family (FOXO1/3/4), PGC-1α, and NF-κB, all of which orchestrate essential cellular responses to redox imbalances. p53 is a central mediator of apoptosis under oxidative stress and is activated by ROS accumulation. Acetylation at K382 enhances proapoptotic transcriptional activity, whereas SIRT1-mediated deacetylation suppresses this function, thereby protecting cancer cells from ROS-induced apoptosis [11]. FOXO transcription factors regulate the expression of antioxidant enzymes, such as SOD2 and catalase. Deacetylation by SIRT1 augments transcriptional activity, enhances antioxidant capacity, and promotes resistance to oxidative damage [12]. PGC-1α acts as a master regulator of mitochondrial biogenesis and oxidative metabolism, and stimulates detoxifying enzymes, such as glutathione peroxidase 1 (GPx1). Deacetylation by SIRT1 enhances mitochondrial efficiency and supports redox balance, particularly under metabolic stress [32]. NF-κB, a pivotal transcription factor in inflammation and SASP, is strongly activated by oxidative stimuli. SIRT1 deacetylates the p65 subunit, thereby reducing pro-inflammatory gene expression and restraining chronic inflammation within the tumor microenvironment [33]. Moreover, elevated NAD^+^ levels under stress conditions further amplify SIRT1 activity and substrate deacetylation, collectively enabling cancer cells to adapt to oxidative, inflammatory, and metabolic stress (Figure 3).

## 4. SIRT1-Mediated Senescence Evasion in Cancer Cells

**Figure 4:**
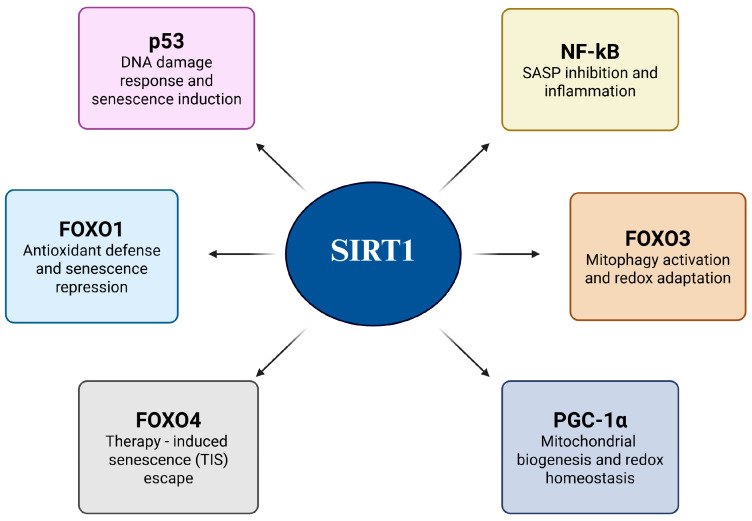
SIRT1-mediated senescence evasion in cancer cells.

Cancer cells evade senescence by reprogramming their redox-sensitive transcriptional networks in response to oxidative stress and genotoxic stimuli. Central to this adaptive mechanism is SIRT1, a NAD^+^-dependent Class III histone deacetylase that fine-tunes transcription factor activity through targeted deacetylation [48]. By selectively removing acetyl groups from key regulators, SIRT1 not only suppresses the expression of senescence- and apoptosis-inducing genes but also reinforces antioxidant defenses and mitochondrial function, thereby establishing a redox-adapted state that favors malignant survival. This dual action underscores the unique role of SIRT1 as both a guardian of cellular longevity and facilitator of oncogenic progression [49]. Figure 4 shows a schematic overview of these processes, illustrating how SIRT1-mediated deacetylation converges through multiple transcriptional pathways to dismantle the senescence barrier. The figure also serves as a conceptual framework for the subsequent substrate-specific analysis (Figure 4 and Table 1).

### 4.1. p53: Suppresses Senescence Induction

The tumor suppressor, p53, is a redox-sensitive transcription factor that initiates cell cycle arrest, apoptosis, and senescence in response to genotoxic stress, oxidative damage, and oncogene activation [50]. One of the key regulatory post-translational modifications of p53 is acetylation at K382 by CBP/p300, which enhances its DNA-binding capacity and transcriptional activation of downstream effectors, such as p21^CIP1, PUMA, and BAX [21,51]. These target genes reinforce senescence and apoptotic responses [52]. SIRT1 is frequently overexpressed in cancer cells and functions as a redox-regulated deacetylase that removes the acetyl group from p53 at K382, thereby repressing its transcriptional activity. This results in the attenuated expression of p21 and pro-apoptotic genes, enabling cancer cells to evade senescence even under persistent DNA damage or oxidative stress [53]. This mechanism is particularly critical in the early stages of tumorigenesis, where oncogene-induced senescence typically acts as a fail-safe mechanism [54]. However, SIRT1-mediated p53 inactivation blocks this barrier, thereby facilitating malignant progression [55]. For instance, in medulloblastoma models, p53 deacetylation is correlated with p21 suppression and escape from senescence [56]. Similarly, in melanoma, defective p53 acetylation impairs p21 induction despite upstream checkpoint activation via CHEK2 [51]. In addition to its canonical role in cell-cycle arrest, acetylated p53 contributes to the regulation of cellular senescence and tumor suppression by influencing inflammatory signaling, immune surveillance, and metabolic homeostasis. Thus, SIRT1-mediated deacetylation of p53 impairs direct growth arrest, suppresses antitumor immunity, and accelerates malignant transformation [57,58].

### 4.2. FOXO1: Represses Senescence While Maintaining Antioxidant Transcription

FOXO1 is a redox-responsive transcription factor that promotes cellular senescence under oxidative stress or DNA damage by upregulating antioxidants, such as manganese superoxide dismutase (MnSOD) and catalase, as well as cell cycle inhibitors, such as p27^Kip1 and GADD45 [31,59]. In response to oncogenic or metabolic stress, FOXO1 contributes to the establishment of a senescent phenotype via its transcriptional activity. However, in cancer cells, SIRT1 reprograms FOXO1 via site-specific deacetylation of lysine residues (e.g., K242, K245, and K262) [60,61]. This post-translational modification induces a functional shift; FOXO1 downregulates pro-apoptotic targets, such as BIM and Fas, while sustaining or even enhancing antioxidant gene expression [31,62]. Consequently, cancer cells can efficiently neutralize intracellular ROS without triggering senescence pathways. Moreover, FOXO1 may indirectly modulate transcriptional regulators, such as AP-1 and BACH2, which are involved in redox adaptation, differentiation, and senescence control [63]. Oncogenic rewiring of the SIRT1–FOXO1 axis has been reported in multiple cancer types, including non-small cell lung cancer (NSCLC) and glioblastoma [64]. Collectively, SIRT1-mediated deacetylation of FOXO1 represents a crucial mechanism by which cancer cells evade senescence and apoptosis, while maintaining redox homeostasis.

### 4.3. FOXO3: Promotes Mitophagy and Redox Adaptation to Evade Senescence

FOXO3 is a redox-sensitive transcription factor activated by oxidative stress, energy deprivation, and DNA damage, and it typically promotes cell cycle arrest and senescence through the induction of p27^Kip1, GADD45, MnSOD, and catalase [65]. However, the SIRT1-mediated deacetylation of FOXO3 at multiple lysine residues (K242, K259, K271, K290, and K569) alters its transcriptional output and nuclear localization in cancer cells [66]. Deacetylated FOXO3 preferentially activates genes involved in antioxidant defense and autophagy, whereas the regulation of cell cycle inhibitors appears to be context-dependent and may vary across cell types and stress conditions [67]. In endometrial cancer, FOXO3 activates BNIP3, which initiates PINK1/Parkin-mediated mitophagy to remove damaged mitochondria and reduce ROS levels [67,68,69]. This process dampens oxidative damage signals and prevents senescence, thereby supporting cancer cell survival, proliferation, and therapeutic resistance. In gastric cancer, a positive feedback interaction between AMPK and FOXO3 has been suggested, which potentially amplifies FOXO3 activity and contributes to enhanced antioxidant defense and metabolic reprogramming [66,70]. Thus, SIRT1-FOXO3-mediated mitophagy and redox homeostasis are key mechanisms by which cancer cells evade senescence [68].

### 4.4. FOXO4: Disrupts p53 Anchoring to Escape Therapy-Induced Senescence

FOXO4 contributes to senescence by forming nuclear complexes with p53, stabilizing it in the nucleus and sustaining p21^CIP1 expression. This FOXO4–p53 interaction is critical for maintaining senescence-associated growth arrest [71,72]. While direct evidence for SIRT1-mediated deacetylation of FOXO4 is limited, K189 has been identified as a lysine residue sensitive to HDAC activity, suggesting possible modulation by Class III HDACs, such as SIRT1 [38]. Disruption of the FOXO4-p53 complex causes p53 translocation to the mitochondria, where it activates caspase-dependent apoptosis. This mechanism is particularly relevant in therapy-induced senescence (TIS), which is triggered by doxorubicin or cisplatin. In cancer cells with elevated SIRT1 activity, destabilization of the FOXO4-p53 complex may facilitate escape from the TIS and promote re-entry into the cell cycle. Recent therapeutic strategies, including FOXO4-DRI peptides and CPP-CAND, aim to selectively disrupt FOXO4-p53 interactions to induce senolysis [72]. Collectively, these results suggest that SIRT1 acts as a molecular switch that determines whether FOXO4-mediated senescence is sustained or bypassed in cancer cells.

### 4.5. PGC-1α: Enhances Mitochondrial Function to Block Senescence Programs

PGC-1α is a transcriptional coactivator that orchestrates mitochondrial biogenesis, oxidative phosphorylation, and antioxidant defense, playing a central role in redox adaptation in cancer cells [38]. Under metabolic stress and excessive ROS, PGC-1α activates genes, such as *NRF1/2*, *TFAM*, *COX IV*, *SOD2*, *GPx1*, and *PRDX3*, thereby sustaining ATP production and detoxifying ROS [39]. PGC-1α activity is enhanced by SIRT1-dependent deacetylation, with lysine 778 (K778) proposed as a potential regulatory site [40]. Enhanced NAD^+^ availability boosts SIRT1 activity, leading to activation of PGC-1α and its downstream partners, including FOXO1, NRF1/2, and ERRα [41]. This transcriptional program suppresses ROS accumulation and DNA damage signaling, thereby attenuating senescence induction via p53- or FOXO-mediated pathways [38,42]. Additionally, PGC-1α has been implicated in modulating the NAD^+^/NADH balance, engaging with the AMPK–SIRT1-FOXO1 axis, and integrating mitochondrial stress responses, supporting cancer cell metabolic flexibility and resistance to therapeutic stress [42]. Thus, the SIRT1–PGC-1α axis constitutes a critical senescence-evasion mechanism by preserving mitochondrial integrity and redox balance in cancer cells, particularly under TIS conditions.

### 4.6. NF-κB: Suppression of SASP and Senescence

NF-κB is a redox-sensitive transcription factor that plays a central role in regulating inflammation, cell survival, and the SASP [73]. Under oxidative stress, NF-κB is activated through the canonical IKK–IκBα signaling cascade, wherein IκBα is phosphorylated and degraded, allowing the p65 (RelA) subunit to translocate into the nucleus [74]. Once in the nucleus, p65 undergoes post-translational modifications that modulate its transcriptional activity [75]. Among these, acetylation at lysine 310 (K310) is particularly critical. It enhances the transcriptional potency of NF-κB without altering DNA-binding affinity, thereby promoting the expression of pro-inflammatory and pro-senescent genes, including IL-6, IL-8, and TNF-α hallmark components of the SASP [76,77]. Persistent oxidative stress in senescent cells leads to sustained K310 acetylation, resulting in chronic NF-κB activation and continuous SASP secretion [78]. This establishes a pro-inflammatory microenvironment that reinforces senescence, induces paracrine senescence in neighboring cells, and paradoxically supports tumor progression and immune evasion.

SIRT1, a NAD^+^-dependent deacetylase activated under oxidative and metabolic stress, counteracts this process by deacetylating p65 at K310 in a redox-sensitive manner. Through this deacetylation, SIRT1 suppresses the transcriptional activity of NF-κB, even in the presence of upstream activating signals, thereby attenuating SASP factor expression and limiting senescence-associated inflammation. Under tumorigenic conditions, the SIRT1-mediated suppression of NF-κB activity confers a selective advantage by enabling senescent evasion and reducing immune surveillance. This contributes to the maintenance of a redox-adapted tumor-permissive microenvironment. Therapeutically, the inhibition of SIRT1 to restore p65 K310 acetylation and reactivate the SASP may represent a promising strategy to trigger tumor-suppressive senescence and enhance the immune-mediated clearance of cancer cells [79].

Recent studies have revealed that SIRT1 not only governs the intracellular redox balance but also plays a key role in shaping the tumor immune microenvironment. Through the deacetylation of transcription factors like FOXO1 and NF-κB, SIRT1 suppresses the expression of pro-inflammatory cytokines and antigen-presenting machinery, thereby promoting immune evasion in cancer. Furthermore, single-cell transcriptomic and redox profiling technologies have revealed significant heterogeneity in SIRT1 expression and redox adaptation among tumor-infiltrating immune cells, especially T-cells and myeloid populations [80]. Notably, redox-resistant immune subpopulations exhibit elevated SIRT1 activity, reinforcing immunosuppression and therapeutic resistance. These insights suggest that targeting the SIRT1–redox axis in defined immune cell subsets may provide promising avenues for combinatorial strategies involving immune checkpoint blockade [81].

## 5. Redox-Dependent SIRT1 Functions Across Cancer Types

**Figure 5:**
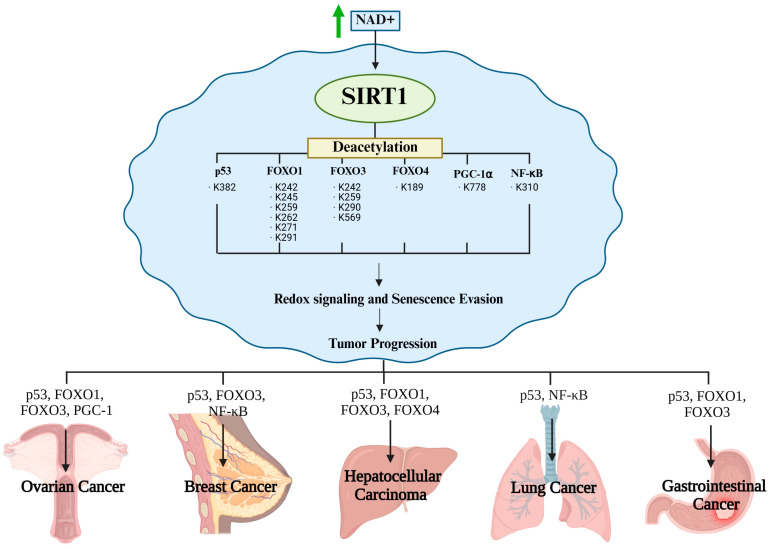
SIRT1-mediated deacetylation promotes cancer cell survival and evasion of senescence. Arrows indicate regulatory relationships and directional flow of the pathway.

SIRT1 is a NAD^+^-dependent deacetylase that supports cancer cell survival by regulating redox-sensitive transcription factors such as p53, FOXO proteins, PGC-1α, and NF-κB. Through these targets, SIRT1 controls key processes, including the suppression of senescence and apoptosis, activation of antioxidant defenses, and regulation of autophagy. These core mechanisms are conserved; however, their outcomes differ across cancer types depending on the redox microenvironment, genetic alterations, and metabolic state [62]. Figure 4 illustrates how SIRT1-driven deacetylation allows tumor cells to evade senescence, withstand oxidative stress, and maintain proliferation under adverse conditions [63]. However, these effects are shaped by tumor-specific features, such as metabolic flexibility, mutational background, and tissue context, resulting in distinct biological consequences. The next section discusses how the redox-modulatory functions of SIRT1 contribute to cancer progression and therapy resistance in a cancer type-specific manner [62] (Figure 5).

### 5.1. Ovarian Cancer: Redox Defense and Aging Resistance

In ovarian cancer, SIRT1 facilitates the cellular adaptation to oxidative stress by modulating key redox-sensitive transcription factors. Deacetylation of p53 at K382 suppresses the transcription of pro-apoptotic and pro-senescent genes, such as p21, PUMA, and BAX, thereby inhibiting cell cycle arrest and apoptosis [82,83]. Deacetylation of FOXO1 and FOXO3 at residues K242, K245, K259, K262, and K291 enhances the expression of antioxidant genes, including MnSOD, catalase, and GADD45, thereby contributing to ROS detoxification [23]. PGC-1α deacetylation promotes mitochondrial biogenesis and oxidative phosphorylation, maintaining energy production and limiting ROS generation [84]. Additionally, SIRT1 upregulates autophagy-related genes, such as LC3 and Beclin-1, promoting autophagy and enhancing resistance to chemotherapeutic stress [85]. According to Peck et al., inhibition of SIRT1 increases p53 acetylation and triggers apoptosis, whereas elevated SIRT1 activity correlates with earlier relapse and reduced sensitivity to ROS-inducing agents in patients with ovarian cancer [86]. Ovarian cancer comprises multiple histological subtypes with distinct origins, molecular features, and clinical characteristics. For example, endometrioid carcinoma is often associated with endometriosis and displays lower-grade malignancy, whereas high-grade serous carcinoma (HGSC) typically originates from the fallopian tube epithelium and is characterized by aggressive growth, extensive genomic instability, and poor prognosis. The function of SIRT1 may differ across these subtypes owing to variations in redox microenvironments, p53 mutation status, and metabolic phenotypes. Future studies should investigate the subtype-specific roles of SIRT1 in redox adaptation and therapy resistance, particularly in HGSC, which is the most lethal form of ovarian cancer [87,88,89]. These findings collectively underscore the contribution of SIRT1 to understanding chemoresistance in ovarian cancer; however, they also highlight critical gaps. Most evidence is derived from cisplatin-resistant cell lines or xenograft models, which may not fully capture the heterogeneity of patient tumors. In addition, contradictory reports on SIRT1 expression across histological subtypes suggest that its role in therapy resistance is not uniform. Although SIRT1 inhibition remains a promising therapeutic strategy, its clinical translation requires validation in patient-derived models and stratification of cohorts according to histological subtype and redox profile

### 5.2. Breast Cancer: Redox Modulation and Senescence Evasion

In breast cancer, SIRT1 promotes tumor progression by enhancing redox adaptation and suppressing senescence. Similar to ovarian cancer, SIRT1 deacetylates p53 at residue K382, which weakens stress-induced apoptotic responses and facilitates tumor survival. [90]. These finding underscores the role of SIRT1 in disabling canonical tumor-suppressive checkpoints, which is a major contribution to our understanding of redox adaptation in breast cancer. However, the evidence is largely based on in vitro models, and it remains uncertain whether similar suppression occurs across heterogeneous patient-derived tumors, particularly those with mutant p53 [88,91]. Deacetylation of FOXO3 at K242, K259, and K271 upregulates antioxidant enzymes, such as SOD2, catalase, and GPx1, thereby maintaining genomic stability under oxidative conditions [92]. In some breast cancer cells, SIRT1 deacetylates NF-κB (p65) at K310, thereby suppressing the transcription of pro-inflammatory and pro-senescent genes, such as IL-6 and IL-8. This contributes to the evasion of SASP-driven senescence and enhances resistance to redox-related therapies [93,94]. Parija et al. reported that SIRT1 inhibition in ERα-positive breast cancer enhances p53 activation and induces apoptosis. SIRT1 also deacetylates ERα, enhancing its transcriptional activity and promoting proliferation in hormone-responsive tumors [95]. Importantly, breast cancer is a heterogeneous disease comprising distinct molecular subtypes, including luminal A/B, HER2-enriched, and triple-negative breast cancer (TNBC), in addition to the classical ER-positive and ER-negative classifications. The function and prognostic implications of SIRT1 may substantially differ across these subtypes owing to their unique redox landscapes, hormone receptor status, and metabolic dependencies. Future studies should elucidate how SIRT1-mediated redox regulation contributes to therapeutic resistance, tumor progression, and immune evasion in each molecular subtype, particularly in aggressive forms, such as HER2-positive and TNBCs. Overall, SIRT1 overexpression in breast cancer is associated with increased malignancy, resistance to oxidative stress, and poor clinical outcomes, positioning SIRT1 as a key modulator of redox signaling and therapeutic escape [88,96]. Collectively, these findings indicate that SIRT1 is a key regulator of redox adaptation and senescence evasion in breast cancer. However, most evidence was from in vitro models or specific subtypes, prohibiting the generalizability to heterogeneous patient tumors. Moreover, conflicting reports suggest that SIRT1 function differs across molecular subtypes, from luminal to HER2+ and TNBC. Thus, while targeting SIRT1 remains promising, future studies must incorporate patient-derived models and stratify them according to the subtype and redox landscape to clarify whether SIRT1 inhibition can provide consistent clinical benefits.

### 5.3. Hepatocellular Carcinoma (HCC): Mitochondrial Redox Control

In HCC, persistent oxidative stress resulting from chronic inflammation, viral infection, and hypoxia contributes to mitochondrial dysfunction and genomic instability [97]. Deacetylation of PGC-1α by SIRT1 enhances mitochondrial biogenesis, improves electron transport chain efficiency, and promotes fatty acid oxidation, thereby reducing mitochondrial ROS production [98]. SIRT1-mediated deacetylation of FOXO1/3 at K242, K259, and K271 induces the expression of antioxidant and DNA repair genes, such as MnSOD, catalase, and GADD45. Deacetylation of FOXO4 at K189 suppresses the transcription of proapoptotic and cell cycle inhibitory genes, thereby promoting cell survival [71,98,99]. SIRT1 modulation of p53 acetylation status, as observed in other cancers, contributes to apoptosis resistance in HCC. In addition, SIRT1 regulates autophagy to support tumor cell survival under metabolic stress [90]. According to Chan et al., pharmacological inhibition of SIRT1 induces apoptosis and reduces the proliferation of HCC cells [100]. Clinically, SIRT1 overexpression is associated with vascular invasion, metastasis, and sorafenib resistance, highlighting its potential as a prognostic biomarker and therapeutic target for aggressive liver cancers [101]. Collectively, these studies support the tumor-promoting role of SIRT1 in HCC through mitochondrial redox control and stress adaptation. However, most data are derived from HBV/HCV-related models, which limit their applicability to NASH- or alcohol-related HCC [100,102]. Furthermore, evidence of the stage-dependent dual role of SIRT1, tumor suppression in early lesions, and oncogenicity in advanced disease, remains underexplored. Therefore, patient cohort studies across different etiologies and disease stages are required to determine whether SIRT1 inhibition can be safely and effectively integrated into liver cancer therapy.

### 5.4. Lung Cancer: Inflammatory Redox Modulation and Immune Evasion

Patients with NSCLC are frequently exposed to chronic oxidative insults arising from environmental factors, such as cigarette smoke, air pollution, and persistent inflammation [103]. These redox stressors drive the sustained activation of NF-κB signaling and promote SASP, which contributes to tumor-promoting inflammation and paracrine senescence in neighboring cells [78]. SIRT1 counteracts this redox-driven proinflammatory state by deacetylating the p65 subunit of NF-κB at lysine 310 (K310), which is essential for full transcriptional activity [93]. Deacetylation at this residue reduces NF-κB’s ability to induce SASP-related cytokines, including IL-6, IL-8, and TNF-α, thereby limiting chronic inflammation and suppressing senescence propagation [78,94]. In addition, in line with the findings in ovarian and breast cancers, SIRT1 suppresses p53 activity in lung cancer cells, aiding in redox adaptation and evasion [22]. Deacetylation of PGC-1α enhances mitochondrial biogenesis and oxidative phosphorylation, stabilizing redox homeostasis under metabolic stress [104]. SIRT1 also upregulates autophagy-related genes, such as LC3 and Beclin-1, promoting survival in hostile microenvironments. Collectively, these redox-adaptive responses foster immune evasion and therapeutic resistance [85]. Studies by Khan and Zhang have demonstrated that pharmacological inhibition of SIRT1 restores p53 and NF-κB acetylation, enhances immune infiltration, reduces metastatic spread, and sensitizes NSCLC cells to chemotherapy [105]. Clinically, elevated SIRT1 expression in NSCLC correlates with a poor response to immunotherapy and shortened patient survival, underscoring its role as a redox-driven regulator of tumor maintenance [105]. Notably, the functional roles of SIRT1 appear to differ among NSCLC histological subtypes. In adenocarcinoma, which often features EGFR or KRAS mutations and relies heavily on mitochondrial oxidative metabolism, SIRT1-driven deacetylation of PGC-1α and FOXO3 plays a central role in maintaining mitochondrial function and redox balance [106,107,108]. These actions support metabolic flexibility and resistance to oxidative stress. In contrast, squamous cell carcinoma, commonly linked to chronic smoking and DNA damage, is more dependent on inflammatory signaling pathways. In this context, SIRT1-mediated suppression of NF-κB activity via deacetylation at K310 may serve as a critical mechanism to restrain SASP-related inflammation and limit paracrine senescence [79,109]. These subtype-specific patterns suggest that SIRT1 supports tumor progression through distinct molecular axes depending on the underlying redox and genetic landscape, warranting tailored therapeutic approaches for each histological subtype. These observations underscore the central role of SIRT1 in the immune evasion and metabolic adaptation of NSCLC cells. However, most mechanistic studies have relied on xenografts, which lack intact immune–tumor interactions. In addition, subtype-specific evidence suggests distinct functions in adenocarcinoma and squamous carcinoma, highlighting context dependency [110,111]. Thus, future studies should use patient-derived and immunocompetent models to clarify whether SIRT1 inhibition can synergize with immunotherapy and define subtype-tailored strategies.

### 5.5. Gastrointestinal Cancer: Mitochondrial Redox Adaptation and Survival Support

In gastrointestinal cancer, SIRT1 enhances tumor cell survival by promoting redox homeostasis, mitochondrial function, and resistance to metabolic stress. SIRT1 regulation of p53-driven transcription is a recurring mechanism across GI tumors that contributes to redox homeostasis and resistance to stress-induced apoptosis [112]. Furthermore, deacetylation of PGC-1α promotes mitochondrial biogenesis and oxidative phosphorylation, ensuring adequate ATP production while minimizing oxidative stress. Under nutrient-deprived and chemotherapeutic conditions, SIRT1 activates autophagy-related genes, such as *LC3* and Beclin-1, supporting cellular adaptation and survival [113,114]. Preclinical studies have shown that pharmacological inhibition of SIRT1 restores p53 acetylation and sensitizes gastrointestinal tumor cells to oxidative stress-induced apoptosis, suggesting that SIRT1 acts as a redox-responsive regulator of tumor progression in gastrointestinal malignancies [56,112]. Collectively, these findings suggest that SIRT1 is a redox-responsive regulator of survival in gastrointestinal cancer. However, the current evidence is predominantly preclinical, with limited patient-based validation. Moreover, contradictory reports have suggested that SIRT1 exerts protective effects in early tumorigenesis by preserving genomic stability. These discrepancies emphasize the need for stage- and tissue-specific analyses to determine whether SIRT1 inhibition is beneficial in advanced GI cancers but potentially detrimental to early lesions [49].

## 6. Therapeutic Targeting of SIRT1–Redox Axis Strategies

**Figure 6:**
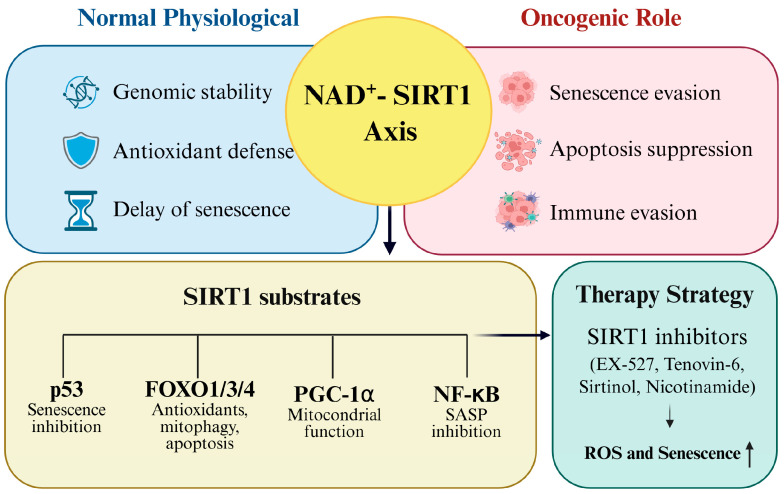
Therapeutic targeting of the SIRT1–redox axis.

As outlined in the preceding sections, SIRT1 enables cancer cell survival by maintaining redox balance and suppressing senescence through the deacetylation of transcription factors, such as p53, FOXO proteins, and NF-κB [62]. Given its central role in oxidative stress adaptation and therapeutic resistance, the pharmacological inhibition of SIRT1 has emerged as a promising approach to disrupt tumor survival mechanisms and enhance the efficacy of existing treatments [82]. Several classes of small-molecule inhibitors, including EX-527, Tenovin-6, Sirtinol, and nicotinamide, have been developed to directly target SIRT1. Although these agents act through distinct mechanisms, such as blocking the NAD^+^ binding pocket or interfering with transcription factor deacetylation, they converge on shared outcomes, such as increased ROS accumulation, apoptosis induction, and reversal of chemoresistance. Notably, some compounds, such as nicotinamide, have advanced to clinical trials (Phase II), underscoring their translational relevance. Figure 6 illustrates the integrated role of the NAD^+^ SIRT1 axis in cancer, highlighting its role in redox regulation, senescence evasion, and therapeutic vulnerability. Table 2 summarizes the representative SIRT1 inhibitors, their mechanisms of action, and their therapeutic effects, demonstrating how pharmacological disruption of this axis can sensitize tumors to oxidative stress and improve treatment responses.

### 6.1. SIRT1 Inhibitors

SIRT1 inhibitors have emerged as promising therapeutic agents for disrupting cancer cell survival and promoting apoptosis [48]. In addition to modulating the cell cycle and apoptotic signaling, many of these inhibitors impair redox homeostasis by restoring the acetylation of transcription factors, such as p53 and FOXO. This leads to increased oxidative stress and sensitizes cancer cells to ROS-mediated cytotoxicity [59]. Several compounds have been developed to target SIRT1, each with a unique mechanism of action and varying degrees of success in preclinical and clinical studies. Below, we discuss the key SIRT1 inhibitors that have shown potential for cancer therapy.

#### 6.1.1. EX-527 (Selisistat)

EX-527, also known as Selisistat, is a highly selective inhibitor of SIRT1. By binding to the NAD^+^ binding pocket of SIRT1, EX-527 blocks deacetylase activity, preventing the regulation of apoptosis and DNA damage responses [115]. This inhibition enhances the activity of tumor suppressor proteins, such as p53, inducing apoptosis and cell cycle arrest in various cancer cell lines, including cervical cancer, melanoma, and chronic lymphocytic leukemia [116]. In addition to its pro-apoptotic effects, EX-527 may impair redox homeostasis by modulating p53 activity, potentially enhancing ROS-mediated cytotoxicity [59]. In ovarian and breast cancers, EX-527 has been shown to reduce chemoresistance by increasing the sensitivity of cancer cells to chemotherapy. Additionally, clinical trials have reported favorable tolerability and initial antitumor activity, particularly in patients with colorectal and breast cancer [117,118]. These findings suggest that EX-527 may be a valuable therapeutic agent for cancers that overexpress SIRT1, particularly in cases of drug resistance.

#### 6.1.2. Tenovin-6

Tenovin-6 is a potent inhibitor of SIRT1 and SIRT2 and enhances the pro-apoptotic function of p53 by increasing its acetylation [119,120]. This dual inhibition provides strong preclinical evidence that Tenovin-6 effectively induces apoptosis, impairs autophagy, and disrupts the redox balance across diverse cancer types. These findings position Tenovin-6 as a prototype compound for the pharmacological targeting of the SIRT axis. Most data are derived from cell-based and xenograft models; therefore, confidence in their clinical translatability remains limited. Furthermore, the fact that Tenovin-6 also inhibits SIRT2 raises concerns about potential off-target effects, particularly considering SIRT2’s physiological roles in normal tissues [121]. While Tenovin-6 disrupts survival pathways, such as Wnt/β-catenin signaling in ALL and autophagy in DLBCL, it remains unclear whether these mechanisms are conserved in solid tumors or reflect cell type-specific vulnerabilities [100,101,102]. Moreover, future investigations should focus on clarifying selectivity, optimizing delivery to minimize systemic toxicity, and determining whether biomarker-driven patient stratification can identify patients who are most likely to benefit.

#### 6.1.3. Sirtinol

Sirtinol is a non-selective inhibitor of both SIRT1 and SIRT2 and affects the acetylation status of key proteins involved in cell cycle regulation and apoptosis [86]. Sirtinol inhibits SIRT1 and increases the acetylation of tumor suppressor proteins, such as p53, thereby inducing apoptosis and autophagic cell death in various cancer cells, including breast cancer [86,122]. Sirtinol demonstrated significant anticancer effects by enhancing oxidative stress and reducing tumor growth in preclinical models [86,122]. These preclinical findings suggest the potential anticancer efficacy of sirtinol. However, further studies are required to evaluate its therapeutic utility and safety in clinical settings.

#### 6.1.4. Nicotinamide

Nicotinamide, the vitamin B3 form, is a potent SIRT1 inhibitor [123]. By inhibiting SIRT1, nicotinamide causes the accumulation of acetylated proteins, which interfere with the cell cycle and promote apoptosis [124]. In breast cancer models, nicotinamide has shown promising results in overcoming drug resistance by disrupting the SIRT1/Akt signaling pathway [125]. Nicotinamide increases ROS accumulation through FOXO and p53 acetylation, linking SIRT1 inhibition to redox imbalance and oxidative stress sensitivity [59]. Nicotinamide is currently being tested in clinical trials, and its ability to sensitize cancer cells to chemotherapy makes it a valuable agent for combination therapies.

### 6.2. Current Trends and Potential of SIRT1 Inhibitors in Cancer Treatment

SIRT1 inhibitors are gaining attention as innovative approaches to cancer therapy. SIRT1 inhibition promotes apoptosis, enhances sensitivity to chemotherapy, and reduces chemoresistance in various cancers. For example, Tenovin-6 induces apoptosis by increasing p53 acetylation, whereas EX-527 exhibits significant anti-cancer effects in ovarian and breast cancer models [117,119]. Several SIRT1 inhibitors, such as EX-527 and nicotinamide, are currently in clinical trials and show promising outcomes in reducing cancer cell proliferation and improving chemotherapy response [126,127]. In addition, SIRT1 inhibitors impair redox homeostasis by restoring the acetylation of transcription factors, such as p53 and FOXO. This results in elevated ROS levels and increased sensitivity to oxidative stress in cancer cells, thereby enhancing their vulnerability to therapeutic interventions. Such redox-disruptive mechanisms help overcome therapeutic resistance, particularly in tumors that rely on adaptive antioxidant responses [59]. More selective and potent SIRT1 inhibitors are currently under development and are expected to further expand their therapeutic potential. Despite the promising results from preclinical and early clinical studies, several challenges remain in the translation of SIRT1 inhibitors into clinical practice [128,129]. First, because SIRT1 also regulates redox balance in normal cells, systemic inhibition may disrupt physiological oxidative homeostasis, potentially leading to off-target toxicity, especially in high-turnover tissues. Second, SIRT1 plays dual roles in cancer, acting as a tumor suppressor in certain contexts (e.g., early-stage cancers or low stress environments) and as a tumor promoter in other contexts (e.g., advanced or redox-stressed tumors). This context-dependent nature complicates broad clinical applications and highlights the need to identify precise molecular signatures that can predict therapeutic benefits. Finally, tumor heterogeneity may lead to variable responses or resistance to SIRT1-targeted therapies [49,96,130]. Mechanisms, such as compensatory activation of alternative NAD^+^-dependent pathways or upregulation of parallel antioxidant systems (e.g., NRF2), may limit therapeutic efficacy. Therefore, rational patient stratification and development of reliable biomarkers are essential for optimizing clinical outcomes. However, translating these findings into clinical benefits remains challenging [131]. First, the dual role of SIRT1 as both a tumor promoter and suppressor, depending on the cellular context, complicates its therapeutic application. Second, most evidence arises from preclinical models that may not fully recapitulate patient tumor heterogeneity or immune–tumor interactions. Third, the lack of validated biomarkers for patient stratification limits the precise identification of patients who will benefit most from SIRT1 inhibition. Finally, systemic toxicity remains a concern, as SIRT1 contributes to redox balance and genomic stability in normal tissues [49,132].

### 6.3. Future Research Directions and Clinical Prospects

Future research into SIRT1 inhibitors has substantial potential for the development of more targeted and effective cancer therapies. Large-scale clinical trials are required to confirm the efficacy and safety of SIRT1 inhibitors in different cancer types. A deeper understanding of how SIRT1 regulates cancer cell survival, proliferation, migration, and drug resistance is essential to optimize therapeutic strategies. In particular, investigating how SIRT1 modulates redox homeostasis and oxidative stress adaptation in cancer cells is critical because redox regulation is closely linked to senescence evasion, metabolic reprogramming, and therapeutic resistance [46,96]. Combining SIRT1 inhibitors with existing cancer therapies, such as immune checkpoint inhibitors, may yield synergistic effects and provide personalized treatment options based on cancer-specific characteristics. Advances in drug delivery technologies may further enhance the specificity and clinical utility of SIRT1 inhibitors by reducing off-target effects and improving therapeutic indices. Importantly, because SIRT1 also contributes to the redox balance and genomic stability in healthy cells, indiscriminate inhibition can result in undesirable toxicity [133]. Therefore, the selective targeting of SIRT1 in cancer cells through tumor-specific delivery systems, context-dependent inhibitors, or exploitation of cancer-specific redox vulnerabilities should be prioritized to maximize therapeutic benefits while minimizing harm to normal tissues. Future research should prioritize the development of cancer-selective SIRT1 inhibitors, the integration of biomarker-driven clinical trial designs, and exploration of rational combination strategies with chemotherapy, immunotherapy, or metabolic modulators [48,133]. Advances in drug delivery, such as nanoparticle-based systems or tumor-targeted prodrugs, may improve specificity and reduce systemic toxicity. Addressing resistance mechanisms such as compensatory NAD^+^ pathways or NRF2 activation is also essential for long-term efficacy [96,128].

## 7. Conclusions

SIRT1 functions as a key molecular regulator that maintains redox homeostasis and suppresses cellular senescence in cancer by deacetylating major transcription factors, such as p53, FOXO, PGC-1α, and NF-κB in a NAD^+^-dependent manner. Through these molecular mechanisms, SIRT1 enables cancer cells to adapt to oxidative stress, evade apoptosis and senescence, and escape immune surveillance.

The SIRT1-mediated redox-protective program includes suppression of senescence- and apoptosis-inducing genes, enhancement of mitochondrial function, activation of antioxidant defense systems, and inhibition of the SASP. Collectively, these adaptations contribute to tumor growth and resistance to anticancer therapies.

Overexpression or hyperactivation of SIRT1 is closely associated with poor prognosis, metastasis, chemoresistance, and oxidative stress resistance in multiple cancer types, including ovarian, liver, breast, and lung cancers. These findings highlight the role of SIRT as a molecular hub linking the metabolic state, redox balance, and senescence regulation within the tumor microenvironment.

Accordingly, therapeutic strategies targeting the SIRT1–redox–senescence axis represent promising approaches for cancer treatment. Inhibitors, such as EX-527, Tenovin-6, and nicotinamide, restore acetylation of p53, FOXO, and NF-κB, reactivating apoptosis and senescence programs, inducing ROS accumulation, and enhancing immune recognition, thereby exerting anticancer effects.

Future research should aim to elucidate the cancer type-specific functions and redox sensitivities of SIRT1, develop predictive biomarkers, and optimize therapeutic targeting to enhance clinical applicability. Furthermore, integrating SIRT1-targeted therapies with immuno-metabolic strategies and deepening our understanding of the NAD^+^–SIRT1–redox axis will pave the way for precision medicine approaches in redox-dependent cancers. Future efforts should emphasize strategies that selectively target SIRT1 in cancer cells while sparing their normal counterparts, thereby improving therapeutic specificity and reducing off-target effects. In summary, although targeting the SIRT1–redox–senescence axis offers promising therapeutic opportunities, its successful clinical translation requires addressing several gaps. These include clarifying SIRT1’s context-dependent roles, developing predictive biomarkers for patient selection, and designing selective delivery systems that can spare normal tissues. Overcoming these challenges is critical to realizing the full potential of SIRT1 inhibition in precision oncology studies.

## Figures and Tables

**Table 1:** SIRT1 substrates and redox-senescence regulatory mechanisms.

Target	Substrate	Critical Regulation Mechanism and Role	Refs.
p53	K382	Deacetylates p53 at K382, repressing p21 and pro-apoptotic genes. Suppresses OIS, immune surveillance, and metabolic arrest to facilitate tumorigenesis.	[21,50,51,52,53,54,55,56,57,58]
FOXO1	K242, K245, K259, K262, K271, K291	Deacetylates FOXO1, suppressing apoptosis but maintaining antioxidant gene expression. Represses AP-1 and promotes BACH2 to prevent senescence.	[31,59,60,61,62,63,64]
FOXO3	K242, K259, K290, K569	Deacetylates FOXO3, reduces p27/GADD45, promotes BNIP3-mediated mitophagy to lower ROS, enabling survival and resistance under stress.	[65,66,67,68,69,70]
FOXO4	K189	Deacetylates FOXO4 at K189, destabilizing the FOXO4–p53 complex, promoting p53 mitochondrial translocation and senolysis. Enables escape from therapy-induced senescence (TIS).	[71,72]
PGC-1α	K778	Deacetylates and activates PGC-1α, enhancing mitochondrial function and antioxidant gene expression (NRF1/2, TFAM). Suppresses ROS and senescence via p53/FOXO blockade.	[38,39,40,41,42]
NF-κB (p65)	K310	SIRT1 deacetylates NF-kB(p65), inhibiting expression of SASP-related cytokines (IL-6, IL-8, TNF-α). This limits chronic inflammation, senescence propagation, and immune recognition in tumors.	[73,74,75,76,77,78,79,80,81]

**Table 2:** Therapeutic compounds based on SIRT1 inhibition in cancer therapy.

Name	Mechanism	Effects	Diseases	Ref.	PubChem CID	Clinical Phase
EX-527 (Selisistat)	Selective SIRT1 inhibitor; blocks p53 repression	Restores p53 acetylation; induces apoptosis; reverses chemoresistance	Breast, Colorectal	[53,115,116,117,118]	10,060,059	
Tenovin-6	Dual SIRT1/2 inhibitor; enhances p53 acetylation	Increases ROS; inhibits Wnt/β-catenin; blocks autophagy	Leukemia	[102,119,120,121]	44,628,478	
Sirtinol	Non-selective SIRT1/2 inhibitor.	Induces apoptosis and autophagy; disrupts redox balance.	Breast	[86,122]	5,311,447	
Nicotinamide	Vitamin B3 derivative; SIRT1 inhibitor.	Increases ROS; induces apoptosis; overcomes drug resistance	Lymphoma	[59,123,124,125]	936	Phase 2

## Data Availability

No new data were created or analyzed in this study. Data sharing is not applicable to this article.
